# Supplementary material for: Utilising Pancreatic Exocrine Insufficiency in the Detection of Resectable Pancreatic Ductal Adenocarcinoma
Source: Cancers (Basel). 2023 Dec 8;15(24):5756. doi: 10.3390/cancers15245756 (PMC10741412; doi:10.3390/cancers15245756)
Supplement: Supplementary file 1 [file cancers-15-05756-s001.zip › Supplementary File S1.pdf]

Calculation of cumulative Percentage Dose Recovered of $^{13}CO_2$

The enrichment of ^{13}C (atom % excess) in breath at time *t* was calculated from the ^{13}C abundance measurements and used, with a measure of the total CO_2 production, to calculate the cumulative percentage dose recovered (cPDR) over 240 minutes, which was expressed as a percentage of the administered ^{13}C -MTG dose as described below:

Percent ^{13}C-dose recovered (PDR) (%/min) = Atom % excess × VCO_2 (mmol/min) × 100/ Dose (mmol)[1]

where Atom % excess = delta over baseline (DOB) × RPDB × 100/1000[2]

DOB = $\partial^{13}C_t - \partial^{13}C_0$ which is defined as the difference between the basal ^{13}C abundance of breath CO_2 before administration of ^{13}C -MTG ($\partial^{13}C_0$) and the ^{13}C-abundance of breath CO_2 at a time point after administration of ^{13}C-MTG ($\partial^{13}C_t$) relative to Vienna Pee Dee Belemnite (PDB). RPDB = 0.0112372 is the $^{13}C/^{12}C$ isotopic ratio of Vienna Pee Dee Belemnite.

VCO_2 is the total expiratory CO_2 production (mmol/h) which was assumed to be 300mmol/min[2] multiplied by the body surface area. The body surface area was calculated by the weight-height formula of Haycock et al[3].

Dose (mmol) is the amount of ^{13}C-MTG administered (mg) / molecular weight of ^{13}C-MTG × n × P/100 where n is the number of ^{13}C atoms per molecule of MTG and P is the ^{13}C isotopic purity (%) of ^{13}C-MTG.

The cPDR over the *n* time points up to time *t* was then calculated using the trapezoidal method[4]:

cPDR over time t =[PDR(n) + PDR(n−1)] / 2 × change in time (min) + cPDR(n−1).

The results of the ^{13}C-MTG breath test were then expressed as the cumulative percentage of ^{13}C-label recovered on breath over 240 minutes ($cPDR_{240}$).

References

1. Herzog DC, Delvin EE, Albert C, et al. 13C-labeled mixed triglyceride breath test (13C MTG-BT) in healthy children and children with cystic fibrosis (CF) under pancreatic enzyme replacement therapy (PERT): a pilot study. *Clin Biochem* 2008; 41: 1489-1492. 2008/09/27. DOI: 10.1016/j.clinbiochem.2008.08.087.
2. Jonderko K, Dus Z, Szymszal M, et al. Normative values for the 13C-mixed triglyceride breath test in two age groups. *Med Sci Monit* 2009; 15: CR255-259. 2009/04/28.
3. Haycock GB, Schwartz GJ and Wisotsky DH. Geometric method for measuring body surface area: a height-weight formula validated in infants, children, and adults. *J Pediatr* 1978; 93: 62-66. 1978/07/01. DOI: 10.1016/s0022-3476(78)80601-5.
4. Matthews JN, Altman DG, Campbell MJ, et al. Analysis of serial measurements in medical research. *BMJ* 1990; 300: 230-235. 1990/01/27. DOI: 10.1136/bmj.300.6719.230.
