# Supplementary material for: Utilising Pancreatic Exocrine Insufficiency in the Detection of Resectable Pancreatic Ductal Adenocarcinoma
Source: Cancers (Basel). 2023 Dec 8;15(24):5756. doi: 10.3390/cancers15245756 (PMC10741412; doi:10.3390/cancers15245756)
Supplement: Supplementary file 1 [file cancers-15-05756-s001.zip › Supplementary File S2.pdf]

Southampton Clinical Trials Unit

DEPEND

Statistical Analysis Plan

**Trial name:**	DEPEND
**Trial registration number:**	C45617/A29908
**Protocol title and version number:**	Protocol version 8 (23rd April 2021)
**SAP version number:**	V1.0
**SAP date:**	27-Oct-2021

To be approved and reviewed by:

	Name	Date
**Chief Investigator**	Zaed Hamady	28/10/2021
**Principal Investigator**	Declan McDonnell	15/11/2021
**Biomedical Scientist**	Paul Afolabi	15/11/2021
**Study Statistician**	Sam Wilding	15/11/2021

UKCRC Registered Clinical Trials Units

Table of Contents

List of Abbreviations 3

1 Introduction 4

1.1 Purpose of SAP 4

1.2 SAP Roles and responsibility 4

1.3 Trial background and rationale (short synopsis) 4

1.4 Objectives 4

1.5 Definition of primary endpoints 5

1.6 Analysis principles 5

2 Design considerations .... Error! Bookmark not defined.

2.1 Description of trial design 5

2.2 Trial power and sample size 5

2.3 Randomisation details 5

2.4 Timing of planned analyses 5

3 Statistical considerations 6

3.1 Definition of analysis populations 6

3.2 Analysis software 6

3.3 Methods for handling data 6

3.4 Definition of key derived variables 6

3.5 General principles for reporting and analysis 7

4 Planned analyses and reporting 8

4.1 Disposition of the study population 8

4.2 Protocol deviations .... **Error! Bookmark not defined.**

4.3 Baseline and demographic characteristics 8

4.4 Compliance to trial drug and treatment .... **Error! Bookmark not defined.**

4.5 Primary endpoints 8

4.6 Secondary endpoints 8

4.7 Additional analyses 8

4.8 Safety reporting 8

5 Tables, listings and figures templates .... Error! Bookmark not defined.

6 References 9

7 SAP revision history 10

List of Abbreviations

Abbreviation		Abbreviation	
^{13}C-MTG	^{13}C Mixed Triglyceride	FE1	Faecal Elastase
PDAC	Pancreatic adenocarcinoma		

1 Introduction

1.1 Purpose of SAP

This statistical analysis plan (SAP) describes in detail the methods that will be used to analyse the data collected as part of the DEPEND study. This will form the basis of the final publication. The final analysis will follow the SAP to ensure that the analyses are conducted in a scientifically valid manner and to avoid post hoc decisions which may affect the interpretation of the statistical analysis. Any deviations from the SAP will be detailed in the final report.

1.2 SAP Roles and responsibility

**Chief investigator:**	**Zaed Hamady**
**Principal investigator:**	**Declan McDonnell**
**Biomedical scientist:**	**Paul Afolabi**
**Study statistician:**	**Sam Wilding**

1.3 Study background and rationale (short synopsis)

The incidence of all types of pancreatic cancer in the UK is 9.6 per 100,000. Pancreatic adenocarcinoma (PDAC) is the most common subtype making up 85% of cases. It is regarded as the most lethal of all the common cancers on account of its insidious nature, often only presenting when causing compression of the distal biliary tree resulting in jaundice; by which point it has usually extended beyond a resectable margin and/or metastasised. This is the case in 79% of pancreatic cancer diagnoses, resulting in a 5-year survival rate of less than 5%; something which hasn't improved in 40 years. Recent studies have shown that around 40% of patients diagnosed with PC require three or more visits to their general practitioner (GP) before they are referred to a specialist thereby leading to a delay in the diagnosis of PC. A recent survey of GPs, showed that not having access to a reliable diagnostic referral pathway is a major barrier to the early diagnosis of PC.

We propose using alternative tests to determine if they are suitable for detecting PDAC in certain populations. This involves the use of a ^{13}C-mixed-triglyceride breath test to test for pancreatic enzyme function and to identify potential lesions which may affect the inner workings of the pancreas. Lipase is an enzyme secreted by the pancreas used in the digestion of fats, and may provide a suitable target for investigating pancreatic function as failure to adequately digest fat could suggest possible duct obstruction or reduced secretory capability due to the presence of a tumour. In addition to this test, faecal elastase can detect patients with insufficient pancreatic function through a single stool sample, as a lower value is associated with poor pancreatic function.

1.4 Objectives

1) Can the ^{13}C-mixed triglyceride breath test and the measurement of faecal elastase-1 be used to distinguish between patients with PDAC and healthy volunteers?
2) Do the 1, 2, and 3-hour ^{13}C-mixed triglyceride breath tests perform similarly in their ability to classify PDAC patients in comparison to the 4-hour test?

1.5 Definition of primary endpoint

1.5.1 Pancreatic adenocarcinoma diagnosis

The primary endpoint for this study is a prediction for stage I or II pancreatic adenocarcinoma (PDAC) vs a prediction of no PDAC.

1.6 Analysis principles

All analyses will be reported according to the TRIPOD Statement

1.7 Description of study design

DEPEND is a case-control prognostic study. Participants with known PDAC will be recruited alongside healthy controls, and a subset of participants with chronic pancreatitis.

1.8 Power and sample size

The sample size calculation was based on a previous study assessing pancreatic exocrine function using a ^{13}C-MTG breath test in healthy subjects and patients with a localised pancreatic mass (Kato *et al*, 1993). Using data from this study, we calculated that a sample size of 25 subjects in each group will give 100% power to detect a difference of 17.8% in the ^{13}C-MTG breath test between patients with PC and healthy subjects assuming a standard deviation of 10.5% in patients with PC at a significance level of 5% using two tailed test. The power calculation was carried out using a statistical software (IBM SPSS Sample Power version 3).

1.9 Randomisation details

DEPEND is a non-randomised study.

1.10 Timing of planned analyses

1.10.1 Interim analyses and early stopping

There are no planned interim analyses or early stopping rules for this study.

1.10.2 Final analysis

The final analysis will be carried out once all participants have had their study visit and the results of their faecal elastase results have been returned.

2 Statistical considerations

2.1 Definition of analysis populations

2.1.1 PDAC population

The PDAC population will consist of 25 participants with a known diagnosis of PDAC.

2.1.2 Healthy control population

The healthy control population will consist of 25 participants with no known diagnosis of PDAC. This population will be age- and sex- matched to those in the PDAC population.

2.1.3 Chronic pancreatitis population

The chronic pancreatitis population will consist of up to 10 participants with a known diagnosis of chronic pancreatitis but no known diagnosis of PDAC.

2.2 Analysis software

The analysis will be conducted using Stata v16 or SAS v9.3, or higher.

2.3 Methods for handling data

2.3.1 Withdrawal from trial

All data up until the point of patient withdrawal from the trial will be used in analyses unless the patient withdrew consent and does not wish for the data already collected prior to withdrawal to be used for the trial.

2.3.2 Missing data

Multiple imputation will be used for key variables with missing data.

2.3.3 Outliers

Patients undergoing the ^{13}C-mixed triglyceride breath test are recommended to remain seated for duration of the test, because physical activity will affect the excretion rate (Kalivianakis *et al*, 1997). Adherence to the protocol will be measured, and additional analyses will be conducted to assess model performance in those who remained seated, and comparing excretion values in compliant and non-compliant participants.

2.3.4 Assumption checking and alternative methods

There are no planned assumption checks in this analysis.

2.3.5 Data transformations

Data transformations are outlined in section 2.4.

2.4 Definition of key derived variables

2.4.1 The cumulative % ^{13}C-dose recovered (cPDR) during exhalation over the study period

After an overnight fast for 12hrs, each participant will be asked to provide 2 baseline breath samples by blowing into 2 × 10ml exetainer tubes through a straw while wearing a facemask before they are given orally 250mg of 2-[^{13}C]-octanoyl-1,3-distearin (^{13}C-MTG -Cambridge Isotope Laboratories, Andover, MA,

USA) together with a solid test meal consisting of 12g of butter on top of a single crispbake and 200ml water. After the test meal is given to each participant, post-prandial breath samples will be then collected every 30mins for 4 hours. After providing breath samples for 4 hours, participants will then be provided with a light meal and then asked to go home. The collected breath samples from the ^{13}C-MTG breath test will be then analysed for the ratio of $^{13}CO_2$ to $^{12}CO_2$ by Dr Paul Afolabi using a Continuous Flow Isotope Ratio Mass Spectrometer (CF-IRMS SERCON Ltd, Crewe, UK) at the Southampton Centre for Biomedical Research Mass Spectrometry (SCBR mass spectrometry). The increase in $^{13}CO_2$ content with regard to the baseline value of the initial breath sample is expressed as atom % excess and we will express the results of the ^{13}C-MTG breath test as the cumulative percentage of ^{13}C-label recovered on breath every 30 minutes until the 4 hour time point (240 minutes).

Normal values for ^{13}C exhalation have been defined in healthy volunteers previously (Keller *et al*, 2014). Participants will be classified as exhibiting pancreatic exocrine insufficiency (PEI) if their cumulative ^{13}C exhalation is below 1.6%, 4.2%, 7.9% or 13.8% at the 1, 2, 3 and 4-hour time points respectively. We will also consider defining cut-offs for PEI based on the mean values for the healthy controls at the respective time points, and the intervening 30-minute time points. We will also examine which cut-off for PEI best discriminates between PDAC and other participants at each time point in this study using the Youden index.

2.4.2 Faecal elastase

Participants will also be asked to provide spot stool samples and FE-1 concentrations will be measured by Chemical pathology service at Southampton General Hospital using a commercially available ELISA kit. Results will be expressed as µg/g of stool. Patients will be provided with a specimen pot in advance of attending for testing so the sample can be delivered to the lab when they attend for the study. Alternatively, a pot can be taken home and then posted back to chemical pathology service when a sample is available.

Cut-offs for FE-1 values indicative of PEI have been established previously (Erikson *et al,* 2008), with values <200µg/g stool indicating PEI and values <100 µg/g stool indicating severe PEI. We will consider the performance of both cut-offs in predicting PDAC. We will consider the inclusion of these cut-offs and the continuous value separately in multivariable logistic regression with cPDR values.

2.4.3 Metabolomic analysis

Serum and plasma samples will be collected, stored and later analysed for each participant to build a profile of proteomics and lipidomics for each cohort to determine if a specific profile exists in those with resectable PDAC compared to the other cohorts.

2.5 General principles for reporting and analysis

95% confidence intervals will be presented throughout the report. Summary statistics will be presented as counts and percentages for categorical variables, or measures of spread for continuous variables (count, mean, standard deviation, median, IQR, minimum, maximum).

2.5.1 Model building

Logistic regression models will be constructed predicting PDAC status using ^{13}C-mixed triglyceride breath test and faecal elastase values as described in section 2.4. Multivariable logistic regression models will then be constructed with the aim of finding the best-fitting combination of cut-offs for the ^{13}C and faecal elastase values.

3 Planned analyses and reporting

3.1 Disposition of the study population

Patient disposition will be summarised in a CONSORT flow diagram, showing a clear account of all patients who entered the study. These will be based on the enrolled population and will include:

- The number of patients assessed for eligibility and reason for not entering study
- The start and stop date of recruitment
- The number of patients in each cohort
- The number of early withdrawals, and reason for withdrawal

3.2 Baseline and demographic characteristics

In order to describe the three cohorts, demographic characteristics will be summarised with counts and percentages or measures of central tendency (mean, SD, median, IQR, range) as appropriate.

3.3 Primary endpoint

3.3.1 Pancreatic cancer diagnosis

The primary endpoint for this study is the prediction of PDAC. PDAC status is known at recruitment (i.e. all participants in the PDAC cohort are positive, all others are negative). Several logistic multivariable models will be constructed using results from the ^{13}C-mixed triglyceride breath test and fecal elastase tests, with appropriate data transformations as outlined in section 2.4. All final regression model results will be reported. Models will be validated and adjusted for optimism using bootstrapping techniques (Steyerberg *et al*, 2001). Performance measures include sensitivity, specificity, positive predictive value, and negative predictive value. In order to evaluate these, numerous cut-offs for defining predicted cancer positivity in the underlying risk score will be evaluated with the study team. Discrimination will be measured using the R2 and D statistic. Calibration will be assessed visually with the aid of calibration plots, comparing observed and predicted risks in each tenth of predicted risk.

3.4 Secondary endpoints

The distribution of ^{13}C cumulative exhalation will be summarised at 1, 2, 3 and 4-hours, by cohort.

The proportion of participants with ^{13}C cumulative exhalation below cut-offs outlined in section 2.4.1 will be summarised by cohort.

The proportion of participants whom remain seated during the 4-hour ^{13}C-mixed triglyceride breath test will be summarised by cohort.

The distribution of the faecal elastase test will be summarised by cohort, including the proportion of participants who returned a sample and whether the sample was collected at the visit or post-visit.

3.5 Additional analyses

No additional analyses will be reported.

3.6 Safety reporting

No safety measures will be reported.

References

For a full list of references, see the protocol.

Erickson JA, Aldeen WE, Grenache DG, Ashwood ER. Evaluation of a fecal pancreatic elastase-1 enzyme-linked immunosorbent assay: Assessment versus an established assay and implication in classifying pancreatic function. Clin Chim Acta. 2008 Nov;397(1-2):87-91

Kalivianakis M, Verkade HJ, Stellaard F, Van Der Werf M, Elzinga H, Vonk RJ. **The ^{13}C-mixed triglyceride breath test in healthy adults: determinants of the $^{13}CO_2$ response**. European Journal of Clinical Investigation, 27: 434-442. https://doi.org/10.1046/j.1365-2362.1997.1310678.x

Kato H, Nakao A, Kishimoto W, Nonami T, Harada A, Hayakawa T, Takagi H. 13C-labeled trioctanoin breath test for exocrine pancreatic function test in patients after pancreatoduodenectomy. Am J Gastroenterol. 1993 Jan; 88(1):64-9.

Keller J, Meier V, Wolfram KU, Rosien U, Layer P. Sensitivity and specificity of an abbreviated (13)C-mixed triglyceride breath test for measurement of pancreatic exocrine function. United European Gastroenterol J. 2014;2(4):288-294. doi:10.1177/2050640614542496

4 SAP revision history

Version number	Revision history	Author	Date
0.1	Created using template	Sam Wilding	21-Apr-2021
0.2	Incorporated changes from Declan McDonnell, taken sample size information from the protocol	Sam Wilding	23-Jul-2021
0.3	Additional information on 13c test added by Paul Afolabi, and additional reference by Declan McDonnell	Sam Wilding	19-Oct-2021
1.0	Escalated to v1.0	Sam Wilding	27-Oct-2021
